# Identification of a PA-Binding Peptide with Inhibitory Activity against Influenza A and B Virus Replication

**DOI:** 10.1371/journal.pone.0007517

**Published:** 2009-10-20

**Authors:** Kerstin Wunderlich, Daniel Mayer, Charlene Ranadheera, Anne-Sophie Holler, Benjamin Mänz, Arnold Martin, Geoffrey Chase, Werner Tegge, Ronald Frank, Ulrich Kessler, Martin Schwemmle

**Affiliations:** 1 Department of Virology, University of Freiburg, Freiburg, Germany; 2 PiKe Pharma GmbH, Zurich, Switzerland; 3 Institute of Pharmaceutical Sciences, Swiss Federal Institute of Technology (ETH), Zurich, Switzerland; 4 Department of Chemical Biology, Helmholtz Centre for Infection Research, Braunschweig, Germany; University of Helsinki, Finland

## Abstract

There is an urgent need for new drugs against influenza type A and B viruses due to incomplete protection by vaccines and the emergence of resistance to current antivirals. The influenza virus polymerase complex, consisting of the PB1, PB2 and PA subunits, represents a promising target for the development of new drugs. We have previously demonstrated the feasibility of targeting the protein-protein interaction domain between the PB1 and PA subunits of the polymerase complex of influenza A virus using a small peptide derived from the PA-binding domain of PB1. However, this influenza A virus-derived peptide did not affect influenza B virus polymerase activity. Here we report that the PA-binding domain of the polymerase subunit PB1 of influenza A and B viruses is highly conserved and that mutual amino acid exchange shows that they cannot be functionally exchanged with each other. Based on phylogenetic analysis and a novel biochemical ELISA-based screening approach, we were able to identify an influenza A-derived peptide with a single influenza B-specific amino acid substitution which efficiently binds to PA of both virus types. This dual-binding peptide blocked the viral polymerase activity and growth of both virus types. Our findings provide proof of principle that protein-protein interaction inhibitors can be generated against influenza A and B viruses. Furthermore, this dual-binding peptide, combined with our novel screening method, is a promising platform to identify new antiviral lead compounds.

## Introduction

Influenza A and B viruses are closely related RNA viruses, which cause respiratory disease in humans and other animals. Both viruses belong to the Orthomyxoviruses, a family of enveloped, negative-sense RNA viruses that encode their genetic information in eight segments. While influenza A viruses (FluA) infect a broad variety of animals as well as humans, influenza B viruses (FluB) are predominantly found in humans [1]. Recently, FluA viruses have attracted the world's attention due to their ability to cause global pandemics, such as the 1918 “Spanish” flu or the novel H1N1 influenza virus of swine origin [2], [3], [4]. However, both FluA and FluB viruses are responsible for seasonal epidemics, which result in hundreds of thousands of deaths each year, since FluB can cause the same spectrum of symptoms that is observed with FluA [5], [6], [7], [8], [9]. In most years, only one type causes the majority of the cases, leading to the exclusion of the others [1]. For this reason, the annual influenza vaccination recommended by the WHO contains both FluA and FluB strains (http://www.who.int/csr/disease/influenza/vaccinerecommendations1/en/). Thus, both types A and B viruses pose a large threat to public health.

Since the current vaccines against influenza viruses offer only incomplete protection, antivirals are greatly needed. Presently, the only class of anti-influenza drugs available which are active against both FluA and FluB are neuraminidase inhibitors oseltamivir (Tamiflu) and zanamivir (Relenza) [10]. However, many influenza A strains, including the circulating H1N1 strains in Europe and the US, are already resistant to oseltamivir [11], [12], suggesting a limited range of use for this type of drug. The resistance is not restricted to FluA, since the emergence of FluB viruses with reduced sensitivity to neuraminidase inhibitors is known [13], [14]. In addition, the treatment of influenza B, particularly in young children, is associated with delayed antiviral effects and clinical resolution [15]. This might be related to the observation that oseltamivir carboxylate, the active metabolite of oseltamivir, is less active against the FluB rather than the FluA virus neuraminidase [13]. Other approved antivirals that target the viral M2 protein, such as amantadine, are only effective against FluA strains, since FluB strains lack a comparable M2 protein [16]. Additionally, the high prevalence of resistance to M2 inhibitors and the general rapid appearance of resistant strains during treatment restricts the therapeutic potential of this class of antiviral drugs [17].

Influenza virus replication and transcription is performed in the nucleus by the heterotrimeric viral polymerase complex consisting of the PB1, PB2, and PA subunits. This includes the synthesis of the genomic RNA from an intermediate copy RNA and the synthesis of viral mRNA transcripts. Synthesis of the latter transcripts is dependent on 8–15 nt long primers derived from the 5′ends of capped endogenous mRNAs. PB1 possesses the RNA polymerization activity, whereas PB2 is known to bind capped mRNA [18], [19], [20]. Recently, RNAse activity could be identified in the N-terminal part of PA, which is most likely necessary to cleave the primer off the remaining host mRNA [21], [22]. The structure of the trimeric complex at the atomic level is not yet resolved, but cryo-EM studies do exist [23], [24]. Recent crystal structures have shown that PB1 binds to PA via a 3_10_-helix at its N-terminus comprised of amino acids (aa) 5–11 [25], [26]. This finding is compatible with previous observations that the PA-binding site is localized to the extreme N-terminus of PB1 [27], [28]. Since the association of these subunits is essential for viral replication [28], [29], [30], [31], and since the sequence of this domain of PB1 is highly conserved among FluA strains [32], this interaction presents an attractive target for antiviral drugs. We have previously shown that a peptide derived from the first 25 amino acids of FluA PB1 can inhibit both the polymerase activity and viral spread of FluA but not FluB [32]. Here, we identify a chimeric FluA/FluB peptide which is active against both virus types.

## Results

### The type-specificity of the PA-PB1 interaction is due to the first 25 amino acids of PB1

**Figure 1:**
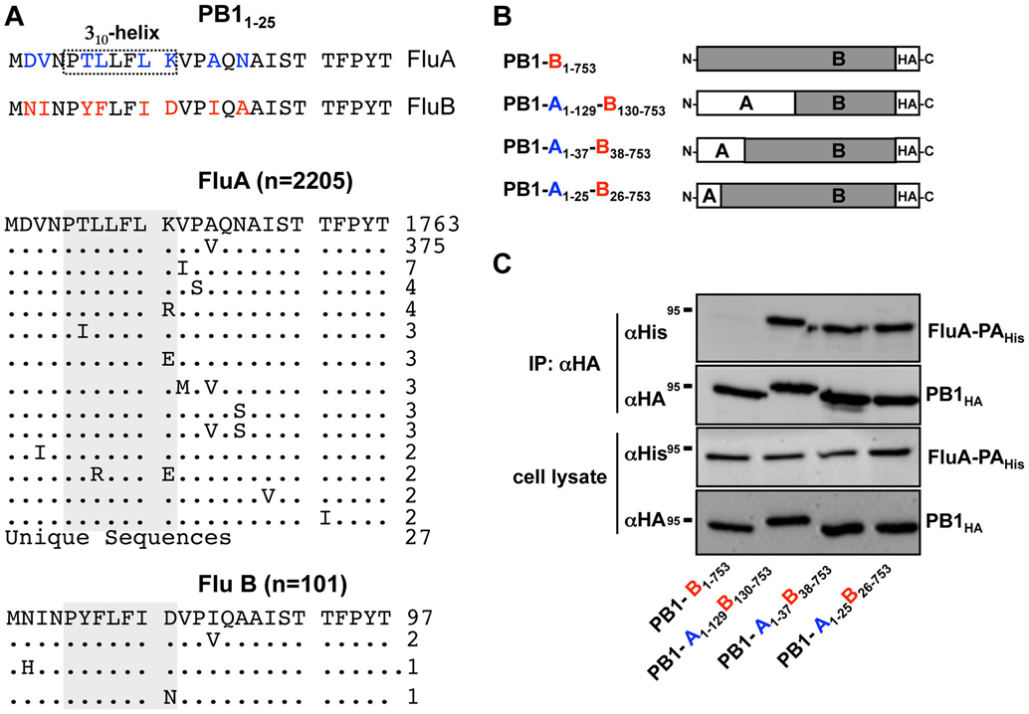
Virus type-specific conservation of the PA-binding domain and interaction of PA with PB1. (A) Upper panel: Alignment of the N-terminal 25 aa of FluA and FluB PB1. The dotted box indicates the 3_10_-helix comprising the core PA-binding domain of PB1. FluA-specific (blue) and FluB-specific (red) aa are highlighted. Middle and lower panels: Alignment of the N-terminal 25 aa of all available FluA and FluB sequences available in the NCBI influenza virus database. Figures on the right hand side indicate the number of sequences present in the database. Grey bars highlight aa which reconstitute the 3_10_-helix of FluA PA and possibly of FluB PA. (B) A/SC35M- and B/Yamagata/73-derived PB1 chimeras used in (b). Note that all PB1 proteins were expressed with C-terminal HA-tags. (C) Human 293T cells were transfected with expression plasmids coding for the indicated PB1 proteins and the C-terminally hexahistidine-tagged PA of FluA (FluA-PA_His_). Cell lysates were prepared 24 hours post transfection and subjected to immunoprecipitation (IP) using anti-HA (αHA) agarose. Precipitated material was separated by SDS-PAGE and analyzed by Western blot for the presence of either His- or HA-tagged polymerase subunits using appropriate antibodies. Protein expression was controlled by analyzing equal amounts of cell lysate. Molecular weights are shown in kilodaltons.

Previously, we have shown that the N-terminal 25 aa of FluA PB1 (PB1_1–25_A) are sufficient to bind FluA-PA and inhibit the polymerase activity of FluA [32]. However, this sequence was not able to block the polymerase activity of FluB. Since the first 25 aa of FluA-PA and FluB-PA differ at 8 positions (Fig. 1A), the failure to block FluB polymerase activity might be due to impaired binding of PB1_1–25_A to FluB PA. Alternatively, the PA-binding domain of FluB PB1 might extend beyond the extreme 25 amino acids. To test this, we generated FluA/B PB1 chimeras by substituting N-terminal regions of varying length from FluA-PB1 into FluB-PB1 (Fig. 1B). After transient co-expression in human 293T cells, we then used immunoprecipitation assays to test which of these chimeras could bind to FluA-PA. All PB1 chimeras tested, including a chimera containing only the first 25 aa of FluA-PB1, could bind FluA-PA equally, whereas wild-type FluB-PB1 could not (Fig. 1C). This implied that the few aa differences in this small domain are the major determinant in PB1 for the type-specific binding of PB1 to PA.

### Quantification of the PA/PB1 interaction

To further characterize which amino acid positions are important for the type-specific binding of PB1_1–25_ to PA, we developed an ELISA-based binding assay to quantify this interaction. For this purpose, we immobilized biotinylated peptides corresponding to PB1_1–25_ on streptavidin-coated plates and subsequently added cell lysates containing HA-tagged PA. Bound PA was detected using HA-specific primary and peroxidase-coupled secondary antibodies. To determine the binding efficiency of variant peptides we performed the ELISA in a competitive manner. In this case, non-biotinylated competitor peptides were added simultaneously with HA-tagged PA to wells containing immobilized PB1_1–25_ peptide.

**Figure 2:**
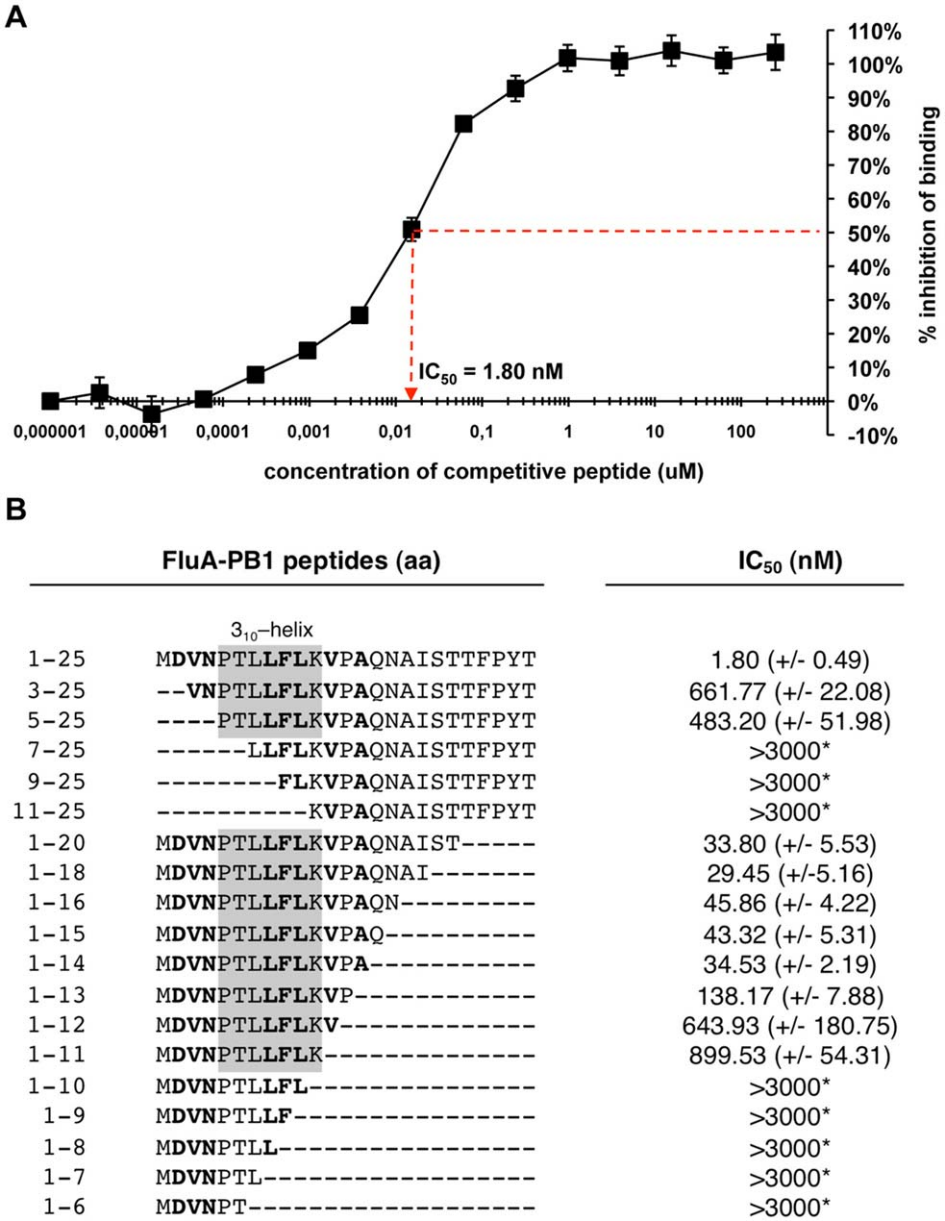
Quantification of the interaction between PB1_1–25_ and PA. (A) Determination of the 50% inhibitory concentration (IC_50_) of PB1_1–25_A by competitive ELISA using the indicated increasing concentrations of peptides and cell extract containing HA-tagged PA of FluA. Error bars represent standard deviations from triplicate experiments. (B) IC50 of PB1_1–25_A-derived peptides. S.D. is shown in parenthesis. Asterisks indicate highest concentrations of peptides (3000 nM) used without detectable inhibitory effect. Grey boxes highlight amino acids that are part of the 3_10_-helix, which was postulated to comprise the core PA-binding region of PB1. Amino acids known to form hydrogen bonds with PA residues are represented in bold.

The core-binding site of the PA-binding domain of FluA-PB1 was recently determined by co-crystalization with the C-terminus of PA and is comprised of a 3_10_-helix ranging from aa 5–11 [25]. We therefore first evaluated our FluA-binding assay by using a soluble wild-type PB1_1–25_A peptide, which resulted in a 50% inhibitory concentration (IC_50_) of 1.8 nM (Fig. 2A). A “scrambled peptide” harboring the same amino acid composition as PB1_1–25_A but with the order randomized, did not block PA-HA binding at highest concentrations (3000 nM, data not shown). Next, the minimal binding domain was determined by deleting aa from the N- and/or C-terminus of PB11-25A. Determination of the IC_50_ using the truncated competitor peptides demonstrated that the 3-10 helix is absolutely essential for binding to PA, since any truncation of this sequence resulted in no competition (Fig. 2B). This is congruent with the results obtained by mutational analysis of the PA-binding domain [28]. Residual competition from partially C- or N-truncated peptides was observed, implying that some of the aa positions flanking the 3–10 helix may be dispensable for binding. Specifically, truncating the C-terminal 10 aa of FluA PB1_1–25_A resulted in an IC_50_ of 43.3 nM compared to 1.8 nM for the full-length peptide (Fig. 2B). Since this truncated peptide can efficiently bind to PA, yet still contains many of the differing aa positions between FluA and FluB, we chose this sequence as a basis to test variant peptides for their binding to both FluA- and FluB-PA.

### A single amino acid substitution allows PB11-25 to bind FluA PA and FluB PA

**Figure 3:**
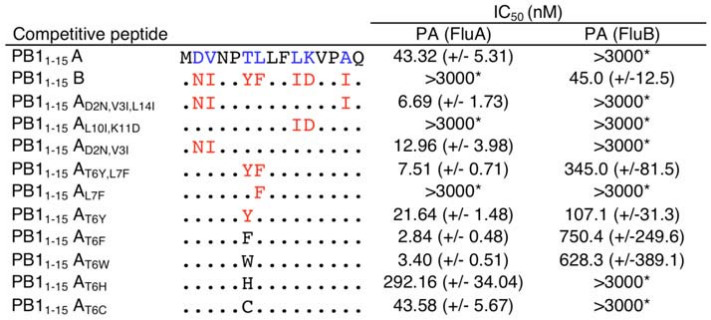
Binding characteristics of FluA and FluB PB1-derived peptide chimera. Inhibitory concentrations of FluA/FluB-derived peptides determined by competitive ELISA. Competitor peptides (0.048 to 3000 nM) were mixed with cell extracts containing HA-tagged PA from either FluA or FluB. Letters in red indicate FluB, letters in blue FluA specific aa. S.D. is indicated in parenthesis. Asterisks indicate highest concentrations of peptides used without reaching 50% inhibition.

As the PB1_1–15_ sequence differs by 8 aa between FluA and FluB (Fig. 1A), we decided to create chimeras containing combinations of FluA- and FluB-specific residues and test these 15mer peptides in the competitive ELISA using both FluA-PA and FluB-PA. For this assay, biotinylated PB1_1–25_A was used in combination with FluA-PA-HA, biotinylated PB1_1–25_B was combined with FluB-PA-HA, and the competitor peptides were tested with both combinations. Competitor peptides PB1_1–15_A and PB1_1–15_B, derived from the wild-type sequences, competed only for FluA PA or FluB PA, respectively, with no cross-type competition detectable (Fig. 3). We then tested competitor peptides containing combinations of FluB-specific aa in the PB1_1–15_A background. While most of these combinations showed no affinity for FluB, substituting positions 6 and 7 led to a significantly reduced IC_50_ for FluB-PA as well as a slight decrease in the IC_50_ for FluA-PA, suggesting that this peptide could bind to both proteins. By substituting only the FluB-specific tyrosine at position 6, efficient binding to PA of both types resulted. In contrast, changing only position 7 of a FluA- to a FluB-specific sequence ablated binding to PA of both types. Introduction of a phenylalanine or tryptophan at position 6 demonstrated that related residues can significantly increase the binding affinity to FluA PA, but only partially increase the affinity to FluB PA. A histidine or cysteine at position 6 led to peptides failing to bind to FluB PA, while binding to FluA PA was still possible, indicating that not all amino acid substitutions confer the dual-binding activity observed with tyrosine at this position.

**Figure 4:**
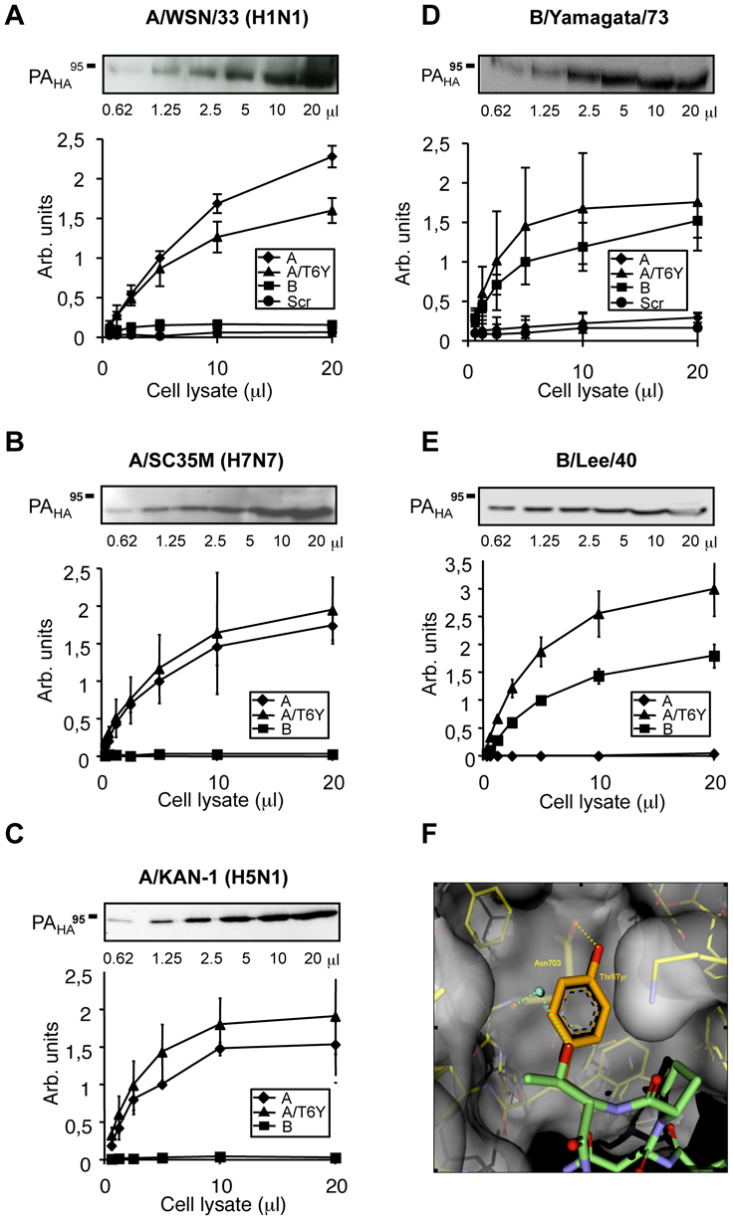
Dual-binding properties of the FluA/B peptide chimera PB1_1–25_A_T6Y_. (A) – (D) Binding of overexpressed HA-tagged PA subunits of differing influenza strains in cell extracts to the immobilized peptides corresponding to the domains of FluA PB1 (PB1_1–25_A), FluB PB1 (PB1_1–25_B) or FluA PB1 T6Y (PB1_1–25_A_T6Y_) was determined by ELISA. Signals using the cognate peptide and lysate were normalized to 1. Binding of the PA subunits to the control peptides was not observed (data not shown). Upper panels: Western blot of the PA-containing cell extracts used. Molecular weights shown in kilodaltons. (F) Structure of FluA PB1_1–15_ (green) bound to FluA PA (grey) as published [25]. T6 forms a hydrogen bond (green line) to a water molecule (blue). Molecular modeling suggests that the aromatic side chain in the mutant T6Y (orange) fits into a hydrophobic pocket and displaces the water molecule.

To further confirm that tyrosine at position 6 of FluA PB1 can bind directly to both FluA- and FluB-PA, we used a biotinylated 25mer peptide PB1_1–25_A_T6Y_ as well as wild-type peptides and cell lysates containing overexpressed PA-HA derived from several different FluA and FluB strains in the ELISA. As shown in Fig. 4A–C, PA-HA from several FluA strains of various subtypes, including a human H5N1 isolate, bound to both PB1_1–25_A and PB1_1–25_ A_T6Y_, but not to PB1_1–25_B or a scrambled biotinylated peptide. Conversely, PA-HA derived from several different FluB strains bound to both the PB1_1–25_B and PB1_1–25_A_T6Y_ (Fig. 4D). Thus, a FluA-PB1-derived sequence containing a single substitution at position 6 can directly bind to PA from different FluA and FluB viruses. Molecular modeling suggests that the FluB-derived tyrosine at position 6 of PB1_1–25_A_T6Y_ fits into a hydrophobic pocket of FluA PA and displaces a water molecule (Fig. 3F). Both the increased hydrophobic interaction as well as the entropic effect of the water displacement might explain the enhanced binding of PB1-T6Y to PA of FluA and possibly of FluB.

### PB_11–25_A_T6Y_ can inhibit the polymerase activity and spread of FluA and FluB

**Figure 5:**
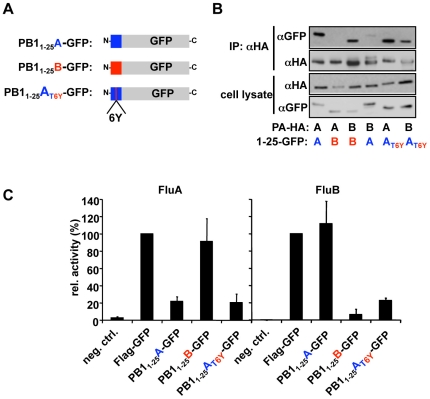
Virus-type independent binding and inhibition of GFP fused to the dual binding peptide PB1_1–25_A_T6Y_. (A) GFP-PB1 fusion proteins used in (B). (B) Complex formation of PB1_1–25_-derived GFP fusion proteins and HA-tagged PA of FluA and FluB. Indicated proteins were expressed in human 293T cells and binding of the GFP fusion proteins was analyzed by IP using anti-HA agarose and subsequent immunoblotting. Precipitated material was visualized using the indicated antibodies for the presence of either HA-tagged PA or GFP. Molecular weights are shown in kilodaltons. (C) Polymerase inhibitory activity of PB1_1–25_-derived GFP fusion proteins in FluA and FluB polymerase reconstitution assays. 293T cells were transiently transfected with a plasmid mixture containing either Flu A (A/WSN/33) or Flu B (B/Yamagata/73) PB1-, PB2-, PA- and NP-expression plasmids, polymerase I (Pol 1)-expression plasmid expressing an influenza virus-like RNA coding for the reporter protein firefly luciferase (FluA) or (FluB) to monitor viral polymerase activity and expression plasmids coding for the indicated GFP fusion proteins. The transfection mixture also contained a plasmid constitutively expressing renilla luciferase (100 ng), which served to normalize variation in transfection efficiency. The activity observed with transfection reactions containing Flag-GFP were set to 100%. The omission of PB2 in the transfection mixture served as a negative control.

To demonstrate that PB1_1–25_A_T6Y_ can bind to both FluA- and FluB-PA in a cellular context, we performed immunoprecipitation experiments using overexpressed PA and PB1_1–25_ peptides fused to GFP (Fig. 5B). This confirmed that PB1_1–25_ derived from FluA or FluB can only bind to FluA-PA or FluB-PA, respectively. However, PB1_1–25_A_T6Y_ could be immunoprecipitated with either FluA-PA or FluB-PA.

Next, we tested the ability of these fusion proteins to inhibit the polymerase activity of both FluA and FluB using a reconstituted viral ribonucleoprotein complex and a reporter gene. As previously shown [32], PB1_1–25_A-GFP inhibits the activity of FluA, but not FluB, while the converse is true of PB1_1–25_B-GFP (Fig. 5C). However, PB1_1–25_A_T6Y_-GFP inhibited both FluA and FluB polymerase activity to comparable levels as the wild-type-derived sequences (Fig. 5C), indicating that this variant peptide is also biologically active.

**Figure 6:**
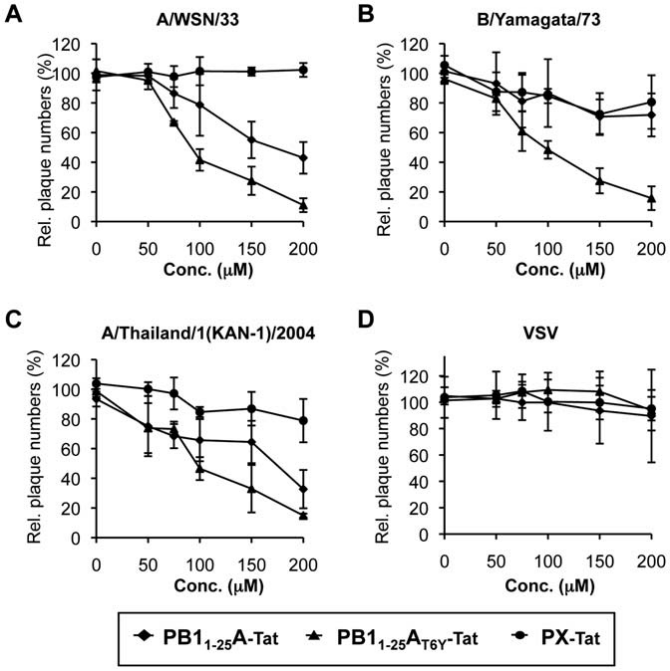
Inhibition of FluA and FluB by the dual binding peptide PB1_1–25_A_T6Y_. Plaque reduction assay using PB1_1–25_A-Tat; PB1_1–25_A_T6Y_-Tat; PX-Tat (control peptide) [32] with FluA, FluB and VSV (vesicular stomatitis virus). A H_2_O control was used to standardize the assay to 100%. Note that PB1_1–25_B-Tat could not be tested due to insolubility. Error bars represent S.D.

Finally, we tested the ability of these peptides to inhibit viral spread in cell culture. MDCK cells were infected with either A/WSN/33 (H1N1), A/Thailand/1/2004 (H5N1), B/Yamagata/73, or vesicular stomatitis virus (VSV) as a specificity control from a different virus family. A plaque reduction assay was then performed in the presence of PB1_1–25_A, PB1_1–25_A_T6Y_, or a negative-control peptide fused to an HIV-Tat derived sequence [33] (Table 1) to allow the peptides to cross the plasma membrane. Similar experiments with a FluB-derived peptide were not possible due to its insolubility. The peptide containing the T6Y substitution inhibited the growth of all influenza strains tested, while PB1_1–25_A-Tat only inhibited FluA (Fig 6). Thus, a single mutation in the PB1_1–25_A sequence results in a peptide which is active against both FluA and FluB strains.

**Table 1:** Peptides used for the plaque-reduction assay.

Peptide name	Peptide sequence
PB1_1–25_A-Tat	MDVNPTLLFLKVPAQNAISTTFPY***TYGRKKRRQRRRPP***
PB1_1–25_A_T6Y_-Tat	MDVNPYLLFLKVPAQNAISTTFPYT****YGRKKRRQRRRPP**
PX-Tat	LSNDELIKKLVTELAENSMIEAEEV***YGRKKRRQRRRPP***

The underlined Y highlights the tyrosine residue derived from the PB1 FluB sequence. Bold letters in italics represent the HIV-Tat derived sequence that allow the peptides to cross the plasma membrane.

## Discussion

Here we report that the PA-binding domains of the polymerase subunits PB1 of influenza A and B viruses are highly conserved within each virus type, and that these domains are not mutually exchangeable. Based on phylogenetic analysis and a biochemical ELISA-based screening approach, we were able to identify a PB1-derived peptide of influenza A virus with a single influenza B-specific amino acid substitution which recognizes PA of both virus types. Significantly, this dual-binding peptide blocked both the polymerase activity and spread of both viruses and raises the exciting possibility of developing new antivirals that specifically interfere with the polymerase complex assembly of both viruses.

The inability of PB1_1–25_A to efficiently bind to FluB-PA and vice versa strongly indicates that these PA-binding domains show limited functional compatibility between the two virus types. We speculate that this incompatibility is caused by an independent evolution of the FluA and FluB polymerase proteins. This is in accordance with a virus-type specific conservation of the PA-binding domain of PB1 (Fig. 1) and further highlighted by an overall aa identity of the viral polymerase subunits of less than 35% between FluA and FluB strains. Together, these differences may account for the failure to reconstitute an active trimeric polymerase complex by mixing FluA and FluB polymerase subunits ([34], [35], [36], unpublished results). However, both FluA and FluB viruses have a common origin [1]. Thus, it is not unexpected that the aa residues of PA which are in close contact with PB1_1–25_ are conserved between FluA and FluB [25], [26], indicating that the PA domain of both virus types bind PB1 in a similar fashion. This similarity might explain how one single amino acid substitution (T6Y) in PB1_1–25_A is sufficient to allow binding to FluB-PA. Based on the available crystal structure of PA complexed with the N-terminus of PB1 and our modeling results with this dual-binding peptide PB1_1–25_A_6Y_ (Fig. 4F), we speculate that the tyrosine residue might exploit a hydrophobic pocket in the PB1-binding domain of FluB PA, thereby stabilizing the interaction. Such an occupation of a hydrophobic pocket may also be important to stabilize the interaction between PB1_1–25_A_6Y_ and FluB PA.

Both the identification of the FluA- and FluB-PA-binding peptide PB1_1–25_A_T6Y_ and our novel ELISA-based screening assay provide a unique platform for future screening of small molecules targeting the PB1/PA interaction of both virus types. Such inhibitors represent attractive new tools to disrupt a variety of protein-protein interactions (PPI) [37], [38]. Initial attempts to identify PPI inhibitors were challenged by the large contact surfaces of protein-protein interfaces. However, the identification of a small subset of aa residues, such as the PA-binding domain of PB1 that contribute to most of the free energy of binding provide excellent targets. Recent screening approaches led to the identification of potent PPI inhibitors that block dimerization between the human protein double mutant 2 (HDM2) and a 15-residue a-helical region of the tumor suppressor p53 [39], the basic helix-loop-helix leucine zipper (bHLHZip) transcription factor Myc and Max [40] or the interaction between paxilin and the integrin a4 [41]. Proof-of-principle that viral protein interactions are also attractive targets for the generation of PPI inhibitors was first obtained with human papilloma virus [42] and herpes simplex virus type 1 (HSV-1) [43]. In the case of HSV-1, the interaction between the catalytic subunit of the HSV-1 polymerase (UL30) and its cofactor, the processivity factor UL42 was targeted. Similar to the interaction of PB1 and PA, the extreme 18 C-terminal residues of UL30 are sufficient to bind to UL42. Using a biochemical screening assay based on the specific interaction of this C-terminal peptide and UL42, several potent small molecule inhibitors were identified [43]. It is therefore reasonable to assume that PPI inhibitors that block the dimerization of PB1 and PA can also be identified using the newly established ELISA-based screening assay. Indeed, recent results indicate that this approach has the potential to identify PPI inhibitors that prevent viral polymerase activity and replication of both types of influenza viruses (Kessler et al., unpublished results). We believe that the PB1/PA interface is an excellent target for PPI inhibitors, because the PA-binding domain within the extreme N-terminus of PB1 is crucial for the interaction with PA, since PB1 mutants lacking this domain fail to interact with PA [28]. Using PB1_1–25_A_6Y_ and FluA PA for screening, we expect that some PPI inhibitors possess a high specificity against both influenza virus types. This is supported by our observations that PB1_1–25_A_6Y_ blocks the growth of FluA and FluB but not VSV.

Besides the PA/PB1 complex, other targets of influenza viruses have become attractive for the development of PPI inhibitors, including the interaction interface between PB2 and PB1, for which the crystal structure was recently solved [44]. Additionally, based on the crystal structure of a fragment of PB2 bound to the cap analog m7GTP [20], non-PPI inhibitors might become feasible that compete with cap-binding properties of PB2. Furthermore, the cap-snatching endonuclease of influenza virus polymerase localized in the N-terminal domain of PA [21], [22] provides a further target for the development of effective inhibitors. However, it has yet to be shown whether these targets are suitable for the development of antivirals. Since the available information of these novel targets is restricted to FluA strains, it remains unclear whether such antivirals may also be active against FluB.

Attempts to substitute position 6 of the FluA PB1_1–25_A peptides with other residues than tyrosine did not result in improved FluB-PA binding in the competitive ELISA experiments. However, replacements with either phenylalanine or tryptophan residues enhanced binding to FluA-PA approximately 20 fold. This suggests that other substitutions may further enhance the binding affinity to FluA-PA and increases the antiviral activity of these peptides. In one study, random substitutions of single aa within an importin-alpha-binding peptide increased its affinity by several orders of magnitude [45], providing proof of principle that the development of potent peptide inhibitors from a peptide lead is possible. The antiviral activity of VIRIP, a peptide that blocks HIV-1 entry by interacting with the gp41 fusion peptide could be increased in its antiretroviral potency by two orders of magnitude [46]. Thus, random substitutions of the PA-binding domain may reveal peptides with significantly enhanced binding properties for FluA-PA. This approach might also lead to the identification of peptides, which bind PA of both virus types, using PB1_1–25_A_6Y_ as the lead peptide. Most importantly, such peptides would be prime candidates for the development of antiviral peptidomimetics.

## Materials and Methods

### Virus strains

Infection experiments were carried out using A/WSN/33 (H1N1) [32] and A/Thailand/1(Kan-1)/2004 [47], B/Yamagata/73 [48] and VSV (serotype Indiana) [49].

### Plasmid constructions

Plasmids pCA-Flag-GFP and pCA-PB1_1–25_A-GFP [32], pCA-PB1-HA [32], the FluA minireplicon plasmids [50] and the expression plasmids for the FluB minireplicon [32] were described elsewhere. The FluB minigenome expression plasmid, pPolI-lucRT_B, was obtained by cloning the firefly luciferase ORF (inverse orientation) flanked by the non-coding region of the segment 8 of the B/Yamagata/73 into the SapI-digested plasmid pPolI-SapI-Rib [50]. For the construction of pCA-PB1_1–25_B-GFP, a linker containing the first 25 codons of PB1 (B/Yamagata/73) was cloned into the EcoRI/NotI sites of pCA-Flag-GFP plasmid, replacing the Flag-coding sequence with PB1_1–25_B. Site directed mutagenesis was carried out with pCA-PB1_1–25_A-GFP to create the plasmid pCA-PB1_1–25_A_T6Y_-GFP. The ORFs of PB1 (B/Yamagata/73) and PA (A/SC35M, A/Thailand/1(KAN-1)/04, A/Vietnam/1203/04, B/Yamagata/73, B/Lee/40; accession numbers: Influenza A virus (A/WSN/1933(H1N1)) segment 2 (CY034138), segment 3 (CY034137), A/SC35M (H7N7) segment 2 (DQ266098), segment 3 (DQ266099), A/Thailand/1(KAN-1)/2004(H5N1) segment 3 (AY555150), B/Lee/40 segment 3 (NC_002206)) were PCR amplified with sense primers containing a NotI site (FluA strains) or a EcoRI site (FluB strains) upstream of the initiation codon and antisense primers with a deleted stop codon followed by an XmaI site, a coding sequence for an HA-tag and a XhoI site. The PCR products were cloned into a modified pCAGGs-vector [51] digested either with EcoRI/XhoI or NotI/XhoI, resulting in pCA-PB1-HA or pCA-PA-HA plasmids, coding for C-terminal tagged versions of the polymerase subunits. To obtain the pCA-PA_A/SC35M_-His plasmid, pCA-PA_A/SC35M_-HA was digested with XmaI/XhoI and the HA coding sequence was replaced by a 6xHis-linker. The A/B-chimeric expression plasmids were obtained by assembly PCR using the pCA-PB1-HA plasmids of SC35M and B/Yamagata/73 and by cloning the resulting PCR product in pCA-PB1_B/Yamagata/73_-HA digested with EcoRI/EcoRV.

### Reconstitution of the influenza virus polymerase activity

293T cells were transiently transfected with a plasmid mixture containing either FluA- or FluB-derived PB1-, PB2-, PA- and NP-expression plasmids, polymerase I (Pol I)-driven plasmid transcribing an influenza A or influenza B virus-like RNA coding for the reporter protein firefly luciferase to monitor viral polymerase activity and with expression plasmids coding for the indicated GFP fusion proteins. Both minigenome RNAs were flanked by non-coding sequences of segment 8 of FluA and FluB, respectively. The transfection mixture also contained a plasmid constitutively expressing *Renilla* luciferase, which served to normalize variation in transfection efficiency. The reporter activity was determined 24 h post transfection and normalized using the Dual-Glu® Lufierase Assay System (Promega). The activity observed with transfection reactions containing Flag-GFP were set to 100%.

### Peptide synthesis

The solid-phase synthesis of the peptides, including the peptides for the plaque reduction assay (Table 1), was carried out on a Pioneer automatic peptide synthesizer (Applied Biosystems, Foster City, USA) employing Fmoc chemistry with TBTU/diisopropylethyl amine activation. Side chain protections were as follows: Asp, Glu, Ser, Thr and Tyr: t-Bu; Asn, Gln and His: Trt; Arg: Pbf; Lys and Trp: Boc. Coupling time was 1 h. Double couplings were carried out if a difficult coupling was expected according to the program Peptide Companion (CoshiSoft/PeptiSearch, Tucson, USA). All peptides were generated as carboxyl amides by synthesis on Rapp S RAM resin (Rapp Polymere, Tübingen, Germany). Biotin was incorporated at the C-terminus of indicated peptides with Fmoc-Lys(Biotin)-OH (NovaBiochem/Merck, Nottingham, UK) and TBTU/diisopropylethyl amine activation for 18 h, followed by coupling of Fmoc-β-Ala-OH for 1 h. Peptides were cleaved from the resin and deprotected by a 3 h treatment with TFA containing 3% triisobutylsilane and 2% water (10 ml/g resin). After precipitation with t-butylmethyl ether, the resulting crude peptides were purified by preparative HPLC (RP-18) with water/acetonitrile gradients containing 0.1% TFA and characterized by analytical HPLC and MALDI-MS. Some peptides were synthesized by peptides&elephants (Nuthetal, Germany) and subsequently purified and characterized as described above.

### Immunoprecipitation experiments

293T cells were transfected with the indicated plasmids in 6-well plates using Metafectene (Biontex, Martinsried, Germany). Cells were incubated 24 h post transfection with lysis buffer (20 mM Tris pH 7.5, 100 mM NaCl, 0.5 mM EDTA, 0.5% NP-40, 1% Protease inhibitor Mix G, (Serva, Heidelberg, Germany), 1 mM DTT) for 15 min on ice. After centrifugation by 13.000 rpm at 4°C supernatant was incubated with anti HA-specific antibodies coupled to agarose beads (Sigma) for 1 h at 4°C. After three washes with 1 ml of washing buffer (lysis buffer without protease inhibitor mix), bound material was eluted under denaturing conditions and separated on SDS-PAGE gels and transferred to PVDF membranes. Viral polymerase subunits and GFP fusion proteins were detected with antibodies directed against the HA- (Covance, Berkeley, California) or His-(Qiagen) or GFP-tag (Santa Cruz Biotechnology).

#### Plaque reduction assay

The experiments were carried out as described [52] with modifications. Confluent MDCK cells were infected with **80 PFU of** A/WSN/33, B/Yamagata/73, A/KAN-1, or VSV/Indiana in PBS at room temperature. After removal of the inoculums, cells were overlaid with medium (DMEM with 20 mM Hepes) containing 1% Oxoidagar and peptides at the indicated concentrations. After incubation for 24 h (VSV), 48 h (A/WSN/33, A/KAN-1) at 37°C with 5% CO_2_, or 72 h at 33°C with 5% CO_2_ (B/Yamagata/73) respectively, cells were fixed with formaldehyde and stained with crystal violet. Plaques were counted and mean plaque number of the water control was set to 100%.

### ELISA

Streptavidin-coated microwell plates were incubated with saturating concentrations of biotinylated peptides, washed and subsequently incubated at room temperature with HA-tagged PA. To obtain PA-HA, 293T cells were seeded into 94 mm-dishes, transfected with the respective plasmid and treated with lysis buffer 24 h post transfection as previously described [32]. After washing the microwell plates, the wells were incubated with an HA-specific primary antibody (Covance, MMS-101R), followed by three washes and an incubation with a peroxidase-coupled secondary antibody (Jackson Immuno Research, Newmarket, UK). After the final wash step, ABTS-substrate (Sigma, ready-to-use solution) was added and the optical density was determined at 405 nm.

The competition ELISA was carried out as described above with the exception that the competitor peptides were added to wells of the plate with bound peptides simultaneously with the addition of the cell extract containing HA-tagged PA subunits.

#### Sequence alignment

Alignments were performed with MUSCLE [53] using the full-length sequences provided from the public influenza virus database (http://www.ncbi.nlm.nih.gov/genomes/FLU/FLU.html).

#### Modeling

Manual docking of the mutated peptide into the PA(C)-PB1(N) crystal structure [25] and subsequent minimization was performed with Accelrys Discovery Studio.
